# Supplementary material for: Dynamic structure of active sites in ceria-supported Pt catalysts for the water gas shift reaction
Source: Nat Commun. 2021 Feb 10;12:914. doi: 10.1038/s41467-021-21132-4 (PMC7876036; doi:10.1038/s41467-021-21132-4)
Supplement: Supplementary file 1 — Supplementary Information [file 41467_2021_21132_MOESM1_ESM.pdf]

Dynamic Structure of Active Sites in Ceria-Supported Pt Catalysts for the Water Gas Shift Reaction

Yuanyuan Li[1,*], Matthew Kottwitz[2], Joshua L. Vincent[3], Michael J. Enright[2], Zongyuan Liu[4], Lihua Zhang[5], Jiahao Huang[1], Sanjaya D. Senanayake[4], Wei-Chang D. Yang[6,7], Peter A. Crozier[3], Ralph G. Nuzzo[2,8], and Anatoly I. Frenkel[1,4]

[1]Department of Materials Science and Chemical Engineering, Stony Brook University, Stony Brook, New York 11794, United States

[2]Department of Chemistry, University of Illinois, Urbana, Illinois 61801, United States

[3]School for Engineering of Matter, Transport and Energy, Arizona State University, Tempe, Arizona 85287-6106, United States

[4]Chemistry Division, Brookhaven National Laboratory, Upton, New York 11973, United States

[5]Center for Functional Nanomaterials, Brookhaven National Laboratory, Upton, New York 11973, United States

[6]Physical Measurement Laboratory, National Institute of Standards and Technology, Gaithersburg, Maryland 20899, United States

[7]Maryland NanoCenter, University of Maryland, College Park, Maryland 20742, United States

[8]Surface and Corrosion Science, School of Engineering Sciences in Chemistry, Biotechnology and Health, KTH Royal Institute of Technology, Drottning Kristinasväg 51, 100 44 Stockholm, Sweden

Supplementary Figures

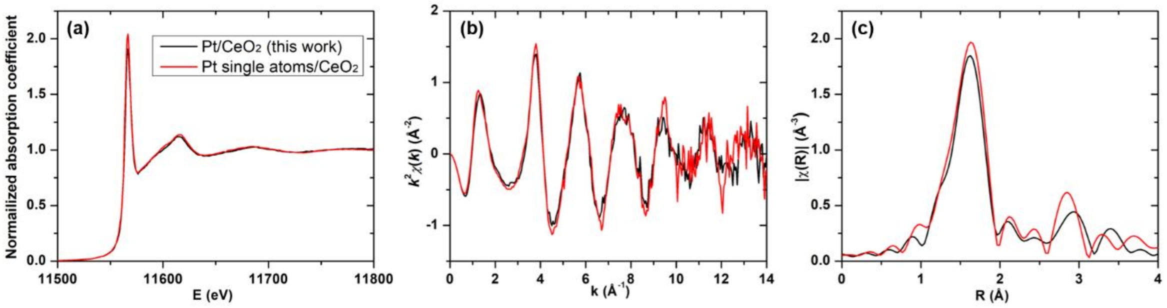

Supplementary Figure 1. Comparison of Pt L_3 edge (a) normalized XANES, (b) k^2 weighted $\chi(k)$, and (c) Fourier transformed $k^2\chi(k)$ spectra of as-prepared Pt/CeO_2 (this work) and ceria supported Pt single atoms.[1]

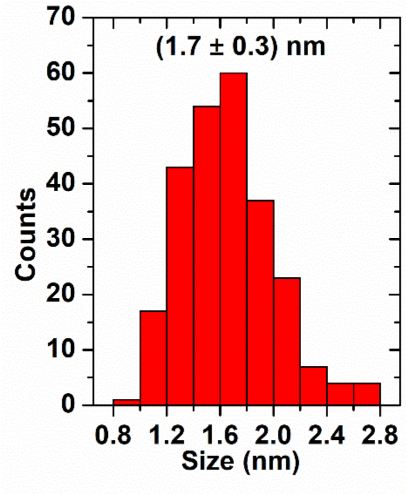

Supplementary Figure 2. The histogram of particle size measured using scanning transmission electron microscopy – annular dark field (STEM-ADF) images, for observed clusters (250 counts) in the reacted Pt/CeO_2 catalyst. The average particle size is about 1.7 nm. The standard deviation is 0.3 nm.

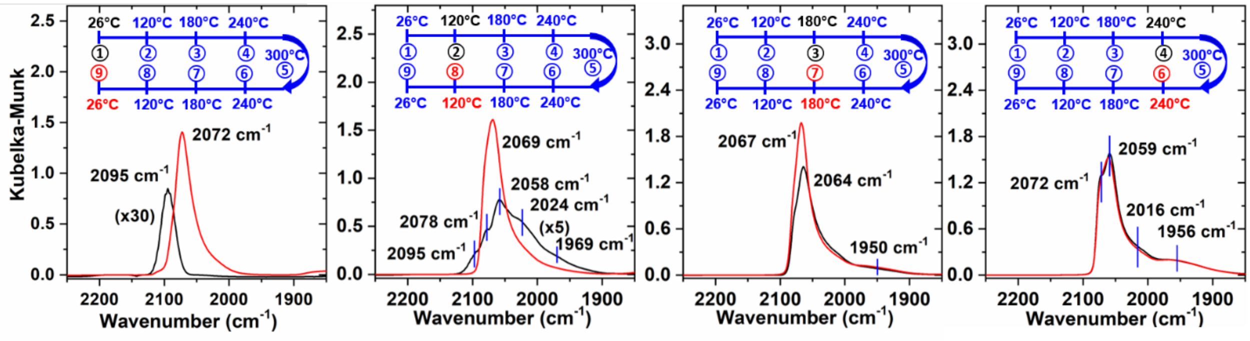

Supplementary Figure 3. The temperature – dependent CO – DRIFTS spectra of Pt supported on ceria. The comparison of CO – DRIFTS spectra collected in the ramp-up and ramp-down process at 26 °C, 120 °C, 180 °C, and 240 °C (left to right). The spectroscopic features (peak position and shape) for species above 180 °C are almost identical between heat-up and cool-down. Similarly, samples at 120 °C and 26 °C during cool down share similar, narrow lineshapes but spectra during ramp-up at 26°C and 120°C are quite different. The as-prepared Pt/CeO_2 at 26°C has a narrow peak centered at about 2095 cm^{-1}, assigned to CO adsorbed on single ionic Pt^{2+} sites.[2] The presence of the sites is confirmed by STEM and XAS (see main text). After heating and cooling, the adsorbed species at 26 °C has a redshifted peak centered at 2072 cm^{-1}, implying CO is adsorbed to a Pt nanocluster.[3] Upon reaching 120 °C during the ramp-up process, the DRIFTS spectrum broadens with several features appearing between 2200-1900 cm^{-1}, suggesting the coexistence of ionic and metallic Pt species on the surface. This mixture of states is not observed at temperatures above 120 °C nor at 120 °C during ramp-down. Instead, a single, narrow cooling peak with the maximum at about 2069 cm^{-1} and with the tail at the low wavenumber side is observed at 120 °C during cooling. The as-prepared, supported Pt single atoms are not stable upon heating up to 180 °C and they aggregate into nanoclusters irreversibly at elevated temperatures. As deduced from the adsorbate vibrational modes, the following valence specific associations can be made. The as-prepared catalysts are exclusively ionic Pt single atoms at room temperature. Upon heating to 120 °C, a mixture of ionic and metallic Pt species coexists, while only metallic Pt species persist at $T \geq 180$ °C. Metallic Pt remains the only predominant species present upon ramp-down to room temperature. These observations corroborate those from the activity test, STEM, and XAS evaluation (Supplementary Fig. 4).

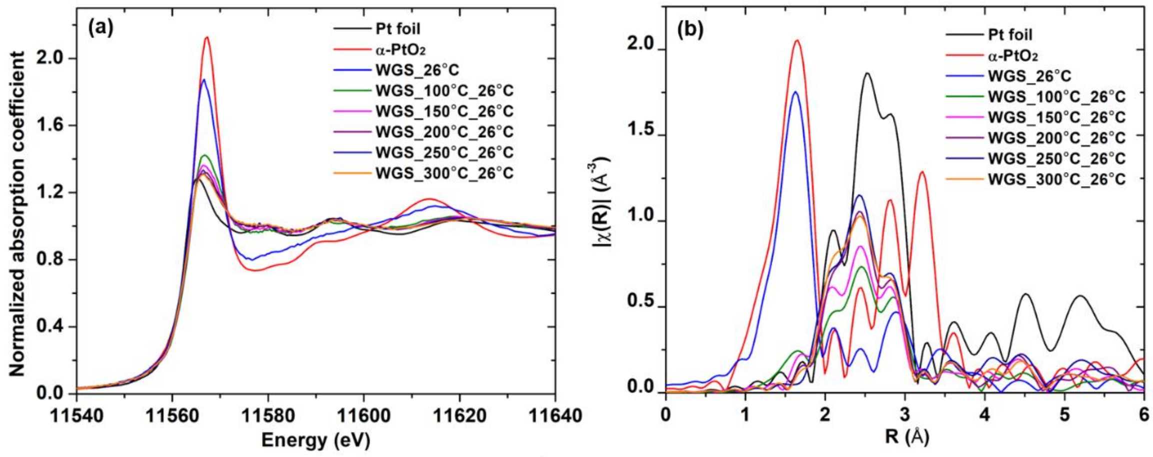

Supplementary Figure 4. The comparison of (a) normalized XANES and (b) Fourier transformed $k^2\chi(k)$ EXAFS spectra of Pt L_3 edge of Pt/CeO_2 under WGS environment at RT (WGS_26°C), after 100°C WGS condition (WGS_100°C_26°C), after 150°C WGS condition (WGS_150°C_26°C), after 200°C WGS condition (WGS_200°C_26°C), after 250°C WGS condition (WGS_250°C_26°C) and after 300°C WGS condition (WGS_300°C_26°C). For

reference, the spectra of Pt foil and α-PtO_2 were included. When T < 200 °C, with the increase of temperature, the catalyst grows from single atoms to nanoclusters. After being treated at 200 °C, 250 °C, and 300 °C, the XAS spectra are similar.

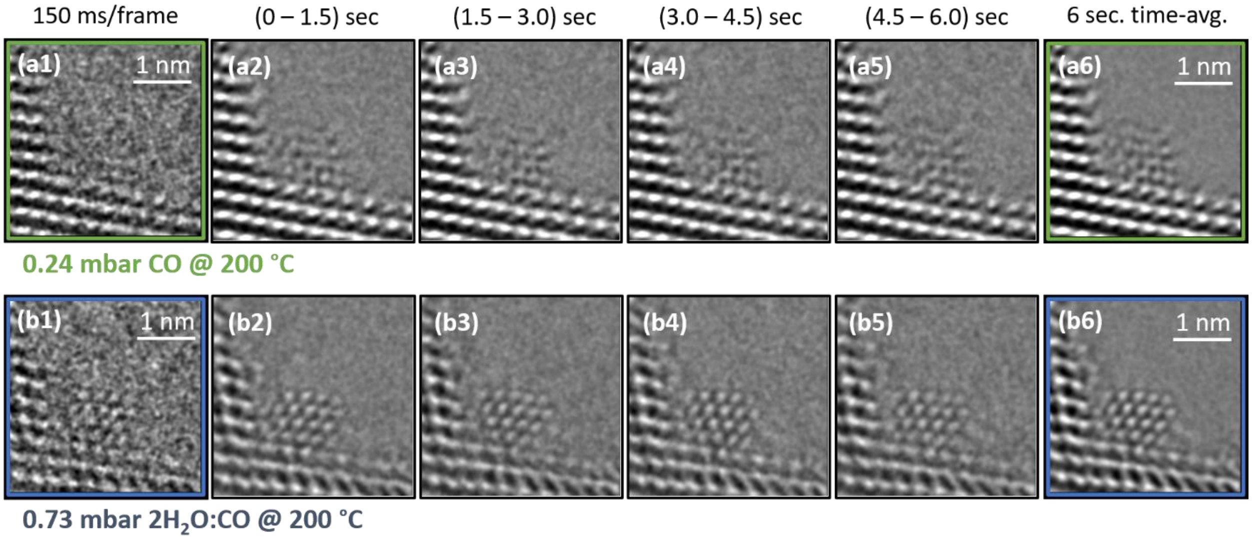

Supplementary Figure 5. In situ ETEM images showing the dynamic structural response and behavior of the Pt/CeO_2 catalyst at 200 °C in CO gas (a1 – a6) and in WGS reaction conditions (b1 – b6). Figures (a1/b1) show the catalyst in a single 150 millisecond frame. Time-averaged image series from sequential 1.5 second intervals are shown in Figures (a2 – a5) and (b2 – b5). Figures (a6/b6) show the time-averaged image over the entire 6 second movie for each condition. The same Pt nanoparticle, which is different from the particle shown in the main text, is shown in all frames. The behavior shown in both figures is representative of many other particles imaged under the same conditions.

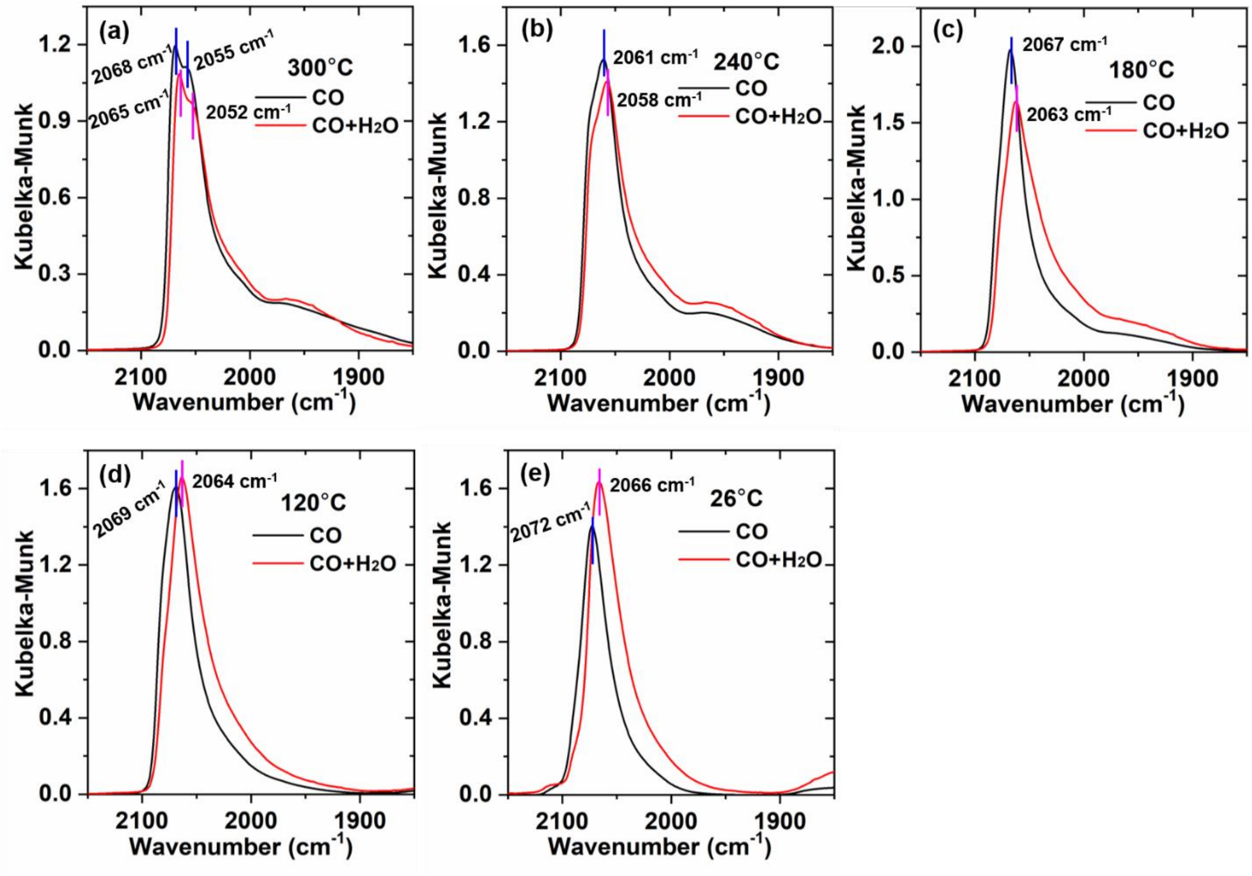

Supplementary Figure 6. At (a) 300 °C, (b) 240 °C, (c) 180 °C, (d) 120 °C, and (e) 26 °C, the changes of CO bands on the Pt surface sites upon the change of condition from CO to WGS. The positions of high frequency peaks were labeled for references.

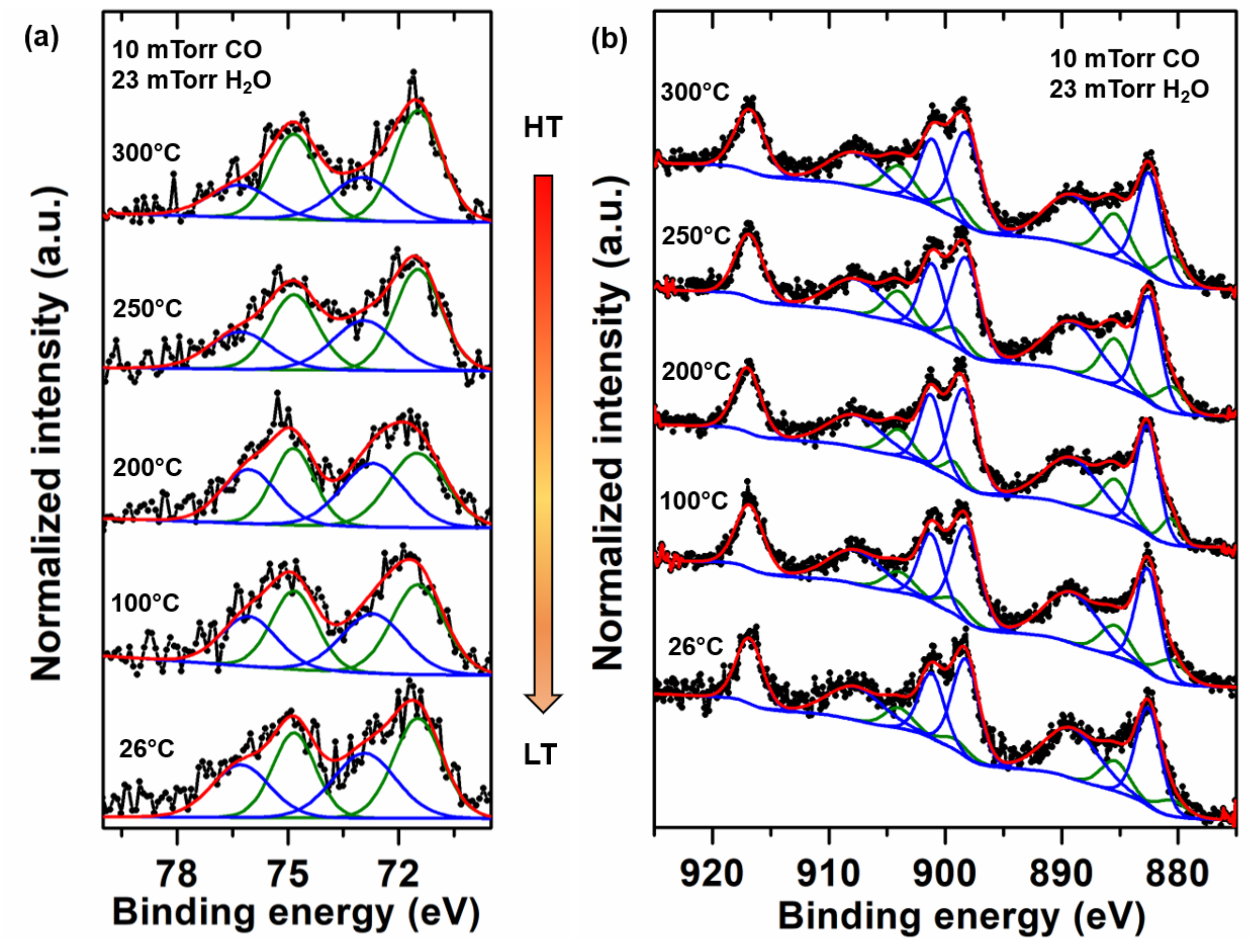

Supplementary Figure 7. The (a) Pt 4*f* and (b) Ce 3*d* region XPS spectra (black) and corresponding fitting spectra (red) for the ceria supported Pt cluster under the WGS reaction conditions at different temperatures. For Pt 4*f* XPS spectra, the data were fitted by Pt^{2+} (blue) and Pt^0 (green) components. For Pt^{2+}, the experimentally observed binding energy of the Pt-4$f_{7/2}$ core level is 72.8 eV and for Pt^0, is 71.5 eV. For Ce 3*d* XPS spectra, the data were fitted by Ce^{4+} (blue) and Ce^{3+} (green) components. For Ce^{4+}, the experimentally observed binding energy of the Ce-3$d_{5/2}$ core level is 882.6 eV and of the Ce-3$d_{3/2}$ core level is 901.2 eV. For Ce^{3+}, the experimentally observed binding energy of the Ce-3$d_{5/2}$ core level is 880.6 eV and of the Ce-3$d_{3/2}$ core level is 899.2 eV.

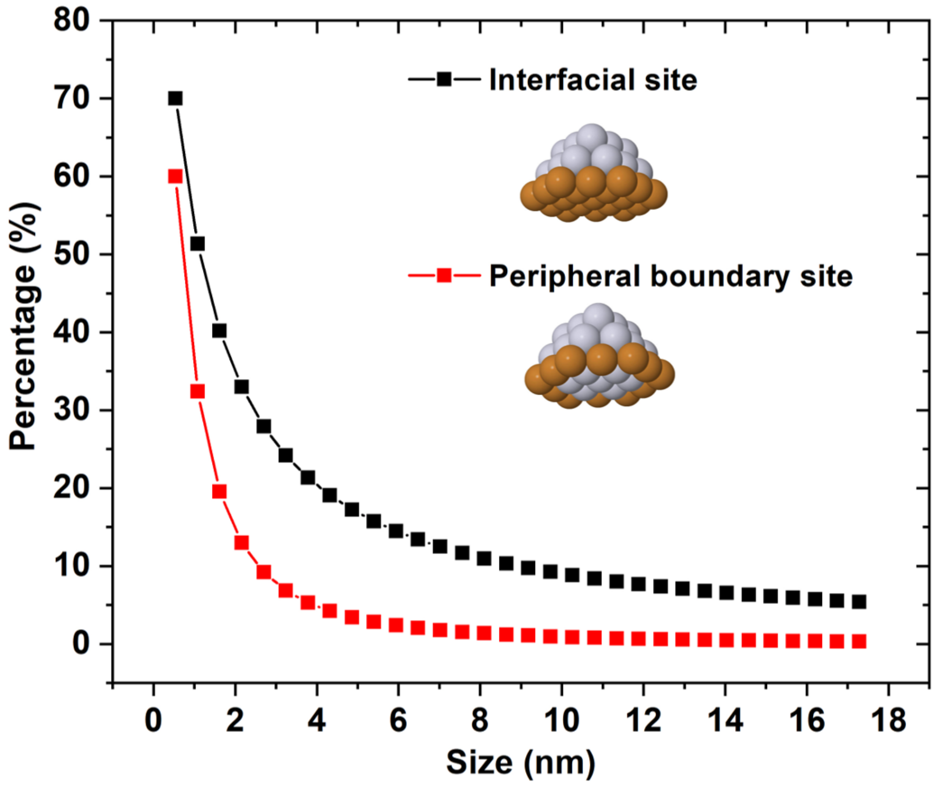

Supplementary Figure 8. The percentage of interfacial and peripheral boundary atoms in the hemispherical cuboctahedron with the size less than 12 nm. For regular polyhedra, the number of interfacial and perimeter atoms could be analytically expressed as the function of the cluster order L. The cluster order L is defined as the number of spacings between adjacent atoms along the edge of the cluster.[4] Assuming the hemispherical cuboctahedron geometry of supported clusters, the number of interfacial atoms in the cluster is $N_i=3L^2+3L+1$, the number of perimeter atoms of the interface is $N_p=6L$, and the total number of atoms in the cluster is $N=5L^3/3+4L^2+10L/3+1$. For the hemispherical cuboctahedron geometry, the particle size could be estimated by using D=2Lr (D is the diameter of the cluster, r is metal-metal bond distance and for Pt-Pt distance, is ≈2.7Å). Then the relationship between the percentage of interfacial/perimeter sites in the particle and the particle site could be made via the cluster order L. Assuming a hemispherical cuboctahedron geometry for a supported nanoparticle, in a 1.7 nm nanocluster, there are approximately 40% atoms at the interface, accounting for the fraction of Pt^{2+} detected by XPS at and below 200 °C.

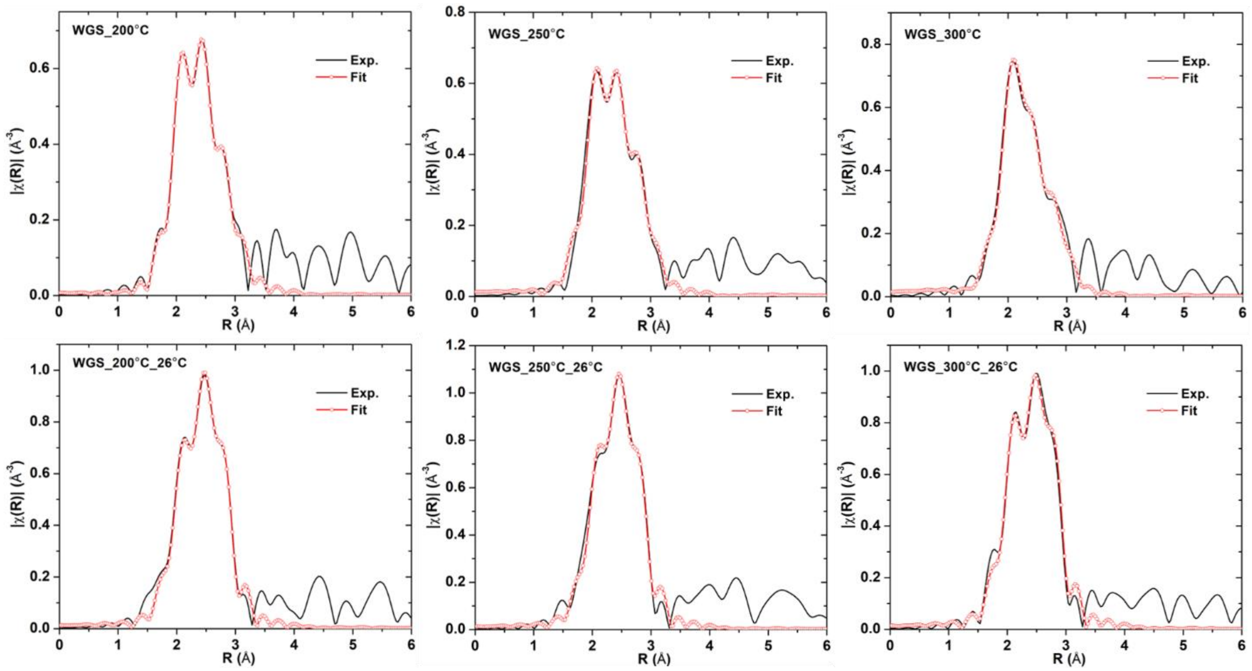

Supplementary Figure 9. The comparison between fitted and experimental spectrum for the XAS data collected at 200°C, 250°C, 300°C and their corresponding post room temperatures under WGS condition. EXAFS data analysis was performed using IFEFFIT package.[5] The amplitude reduction factor S_0^2 was obtained by fitting the spectrum of Pt foil measured at the same beamline. The value of S_0^2 (0.83) was used for all subsequent fitting the spectra of Pt/CeO_2. In fitting the spectra of Pt/CeO_2, two nearest-neighboring photoelectron paths chosen in the fitting model are Pt-O and Pt-Pt. For all data, the fitting k range is 2.8 $Å^{-1}$ to 14.0 $Å^{-1}$ and the fitting R range is 1.6 Å to 3.2 Å. The best fitting results are summarized in Supplementary Table 2.

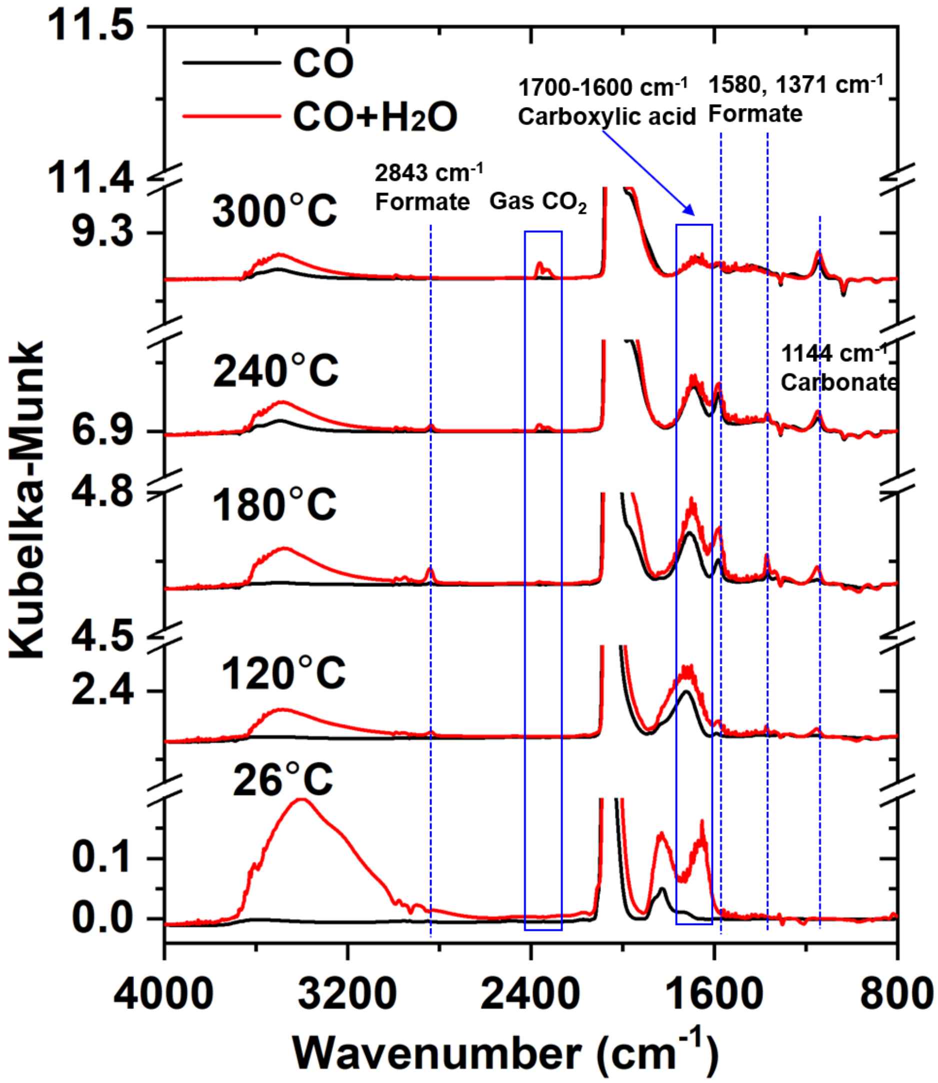

Supplementary Figure 10. The DRIFTS spectra at different temperatures (ramp-down process) under WGS and CO conditions for Pt/CeO_2 catalyst. The assignments to the peaks are based on the literature.[6-9] We note here that the existence of carbon-containing intermediates (formate, carbonate, carboxylic acid) suggests that the associative mechanism may also play role. However, to identify or discriminate active/spectator species in the WGS reaction, advanced transient isotopic experiments are needed.

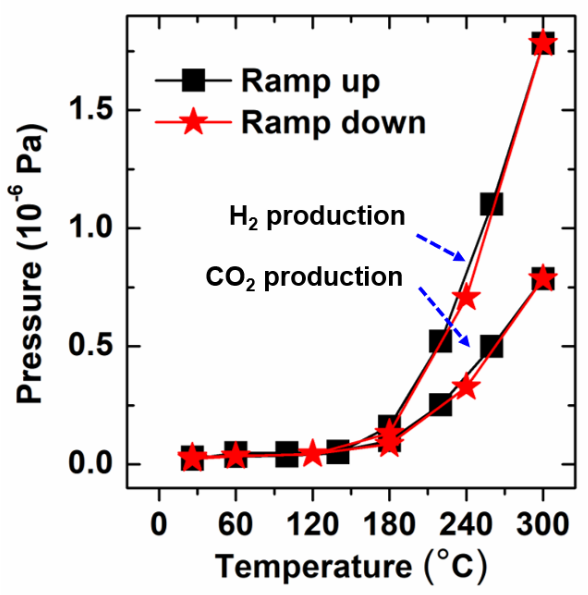

Supplementary Figure 11. Activity test using the DRIFTS reactor: the H_2 and CO_2 productions at different temperatures in the ramp up and down processes. Similar trend was observed using the Clausen cell (Fig. 1(a)).

Supplementary Tables

Supplementary Table 1. Relative concentration of Pt (4*f*) and Ce (3*d*) measured by AP-XPS

Condition	Pt %	Ce %
WGS_300°C	9.87	90.13
WGS_250°C	9.76	90.24
WGS_200°C	11.49	88.51
WGS_100°C	11.27	88.73
WGS_26°C	13.97	86.03

Supplementary Table 2. The best EXAFS fitting results of coordination numbers (N), bond distances (R), disorder factors (σ^2) and energy shifts (ΔE_0) for the data collected under different conditions. The errors were obtained by performing analysis with IFEFFIT package.[5]

Condition	Bond	N	R(Å)	σ^2 (Å^2)	ΔE_0 (eV)	*C_3 (Å^3)
WGS_200°C	Pt-O	1.4±0.3	2.57±0.01	0.000±0.002	8.1±1.3	
	Pt-Pt	5.9±0.3	2.78±0.01	0.0073±0.0009	4.9±1.3	0.0006±0.0002

WGS_200°C _RT	Pt-O	1.2±0.4	2.65±0.02	0.001±0.002	13.8±1.5	
	Pt-Pt	7.9±0.8	2.746±0.004	0.0068±0.0005	2.9±0.7	
WGS_250°C	Pt-O	1.4±0.6	2.55±0.02	0.001±0.004	10.4±2.7	
	Pt-Pt	8.5±1.0	2.76±0.03	0.009±0.002	2.6±2.4	0.0005±0.0004
WGS_250°C_RT	Pt-O	1.3±0.4	2.65±0.01	0.000±0.002	13.8±1.5	
	Pt-Pt	8.4±0.8	2.741±0.003	0.0068±0.0005	2.9±0.7	
WGS_300°C	Pt-O	2.2±0.3	2.53±0.01	0.000±0.003	9.8±1.2	
	Pt-Pt	9.1±0.7	2.76±0.02	0.010±0.002	0.8±1.2	0.0009±0.0003
WGS_300°C_RT	Pt-O	1.3±0.4	2.61±0.01	0.000±0.002	13.8±1.5	
	Pt-Pt	8.5±0.9	2.736±0.004	0.0068±0.0005	2.9±0.7	

* For small clusters at high temperatures, due to vibrational anharmonicity, the third cumulant (C_3) should be considered in the fitting to improve accuracy in the bond distance determination.)

Supplementary References

1. Kottwitz, M. *et al.* Local structure and electronic state of atomically dispersed Pt supported on nanosized CeO_2. *ACS Catal.* **9**, 8738-8748 (2019).
2. Nie, L. *et al.* Activation of surface lattice oxygen in single-atom Pt/CeO_2 for low-temperature CO oxidation. *Science* **358**, 1419-1423 (2017).
3. Ding, K. *et al.* Identification of active sites in CO oxidation and water-gas shift over supported Pt catalysts. *Science* **350**, 189-192 (2015).
4. Montejano-Carrizales, J. M., Aguilera-Granja, F. & Morán-López, J. L. Direst enumeration of the geometrical characteristics of clusters *Nanostruct. Mater.* **8**, 269-287 (1997).
5. Ravel, B. & Newville, M. ATHENA, ARTEMIS, HEPHAESTUS: data analysis for X-ray absorption spectroscopy using IFEFFIT. *J. Synchrotron Radiat.* **12**, 537-541 (2005).
6. Li, C. *et al.* Adsorption of carbon monoxide and carbon dioxide on cerium oxide studied by Fourier-transform infrared spectroscopy. Part 2.—Formation of formate species on partially reduced CeO_2 at room temperature. *J. Chem. Soc., Faraday Trans.1* **85**, 1451-1461 (1989).
7. Bazin, P., Saur, O., Lavalley, J. C., Daturi, M. & Blanchard, G. FT-IR study of CO adsorption on Pt/CeO_2: characterisation and structural rearrangement of small Pt particles. *Phys. Chem. Chem. Phys.* **7**, 187-194 (2005).
8. Li, C. *et al.* Carbon monoxide and carbon dioxide adsorption on cerium oxide studied by Fourier-transform infrared spectroscopy. Part 1.—Formation of carbonate species on dehydroxylated CeO_2, at room temperature. *J. Chem. Soc., Faraday Trans.1* **85**, 929-943 (1989).
9. Pozdnyakova, O. *et al.* Preferential CO oxidation in hydrogen (PROX) on ceria-supported catalysts, part I: oxidation state and surface species on Pt/CeO_2 under reaction conditions. *J. Catal.* **237**, 1-16 (2006).
