# Supplementary material for: Dynamic structure of active sites in ceria-supported Pt catalysts for the water gas shift reaction
Source: Nat Commun. 2021 Feb 10;12:914. doi: 10.1038/s41467-021-21132-4 (PMC7876036; doi:10.1038/s41467-021-21132-4)
Supplement: Supplementary file 3 — Description of Additional Supplementary Files [file 41467_2021_21132_MOESM3_ESM.pdf]

Description of Additional Supplementary Files

File Name: Supplementary Movie 1
Description: Environmental TEM image time-series of a CeO_2-supported Pt nanoparticle in 0.24 mbar CO gas at 200 °C. The movie is 6 seconds long with 150 milliseconds per frame. The images have been aligned and processed with a 2-pixel Gaussian blur for clarity.

File Name: Supplementary Movie 2
Description: Environmental TEM image time-series of a CeO_2-supported Pt nanoparticle in 0.73 mbar $2H_2O$:CO gas at 200 °C. The movie is 6 seconds long with 150 milliseconds per frame. The images have been aligned and processed with a 2-pixel Gaussian blur for clarity.
